# Supplementary material for: Smartphone scanning is a reliable and accurate alternative to contemporary residual limb measurement techniques
Source: PLoS One. 2024 Dec 2;19(12):e0313542. doi: 10.1371/journal.pone.0313542 (PMC11611122; doi:10.1371/journal.pone.0313542)
Supplement: S1 Table — Numbers enclosed in brackets indicate standard error measurements. Unfortunately the data for Polycam Web in the Studio, Office and Outdoors environments were lost. (PDF) [file pone.0313542.s001.pdf]

**Capture Location**	**Change in Mean**				
	**Polycam**	**Polycam** *Web*	**Luma**	**Luma** *Web*	**Meshroom**
Studio	-0.4 (0.2)	N/A	0.2 (0.4)	-0.1 (0.1)	-0.4 (0.3)
Office	-0.9 (1.3)	N/A	0.1 (0.3)	-0.3 (0.3)	0.0 (0.4)
SportsHall	0.3 (0.7)	N/A	-0.1 (0.1)	-0.3 (0.4)	-0.9 (0.1)
Outdoors	0.1 (0.3)	N/A	0.3 (0.3)	0.0 (0.1)	-0.3 (0.4)
Home(Natural)	-1.6 (1.0)	-0.2 (0.1)	0.3 (0.1)	-0.6 (0.5)	-0.7 (0.6)
Home(Artificial)	-0.9 (0.2)	-1.1 (0.7)	-0.5 (0.1)	-1.6 (0.4)	-0.9 (0.4)
Home(Point)	-2.5 (1.0)	-6.0 (1.0)	-0.1 (0.0)	-1.9 (0.9)	-57.6 (16.4)
Average	-0.8 (0.7)	-2.4 (0.6)	0.0 (0.2)	-0.7 (0.4)	-8.7 (2.6)
