# Supplementary material for: Smartphone scanning is a reliable and accurate alternative to contemporary residual limb measurement techniques
Source: PLoS One. 2024 Dec 2;19(12):e0313542. doi: 10.1371/journal.pone.0313542 (PMC11611122; doi:10.1371/journal.pone.0313542)
Supplement: S1 Fig — Left: RMSE values across a transtibial (Participant C), transradial (Participant E), transhumeral (Participant E), and transfemoral (Participant A), for different photoset sizes. Sampled photo-set sizes include 25, 36, 50, 75, 100, 125, and 150 photographs. Right: Signed distances across the same limbs, and their maximum and minimum deviations from the criterion surface. (PDF) [file pone.0313542.s002.pdf]

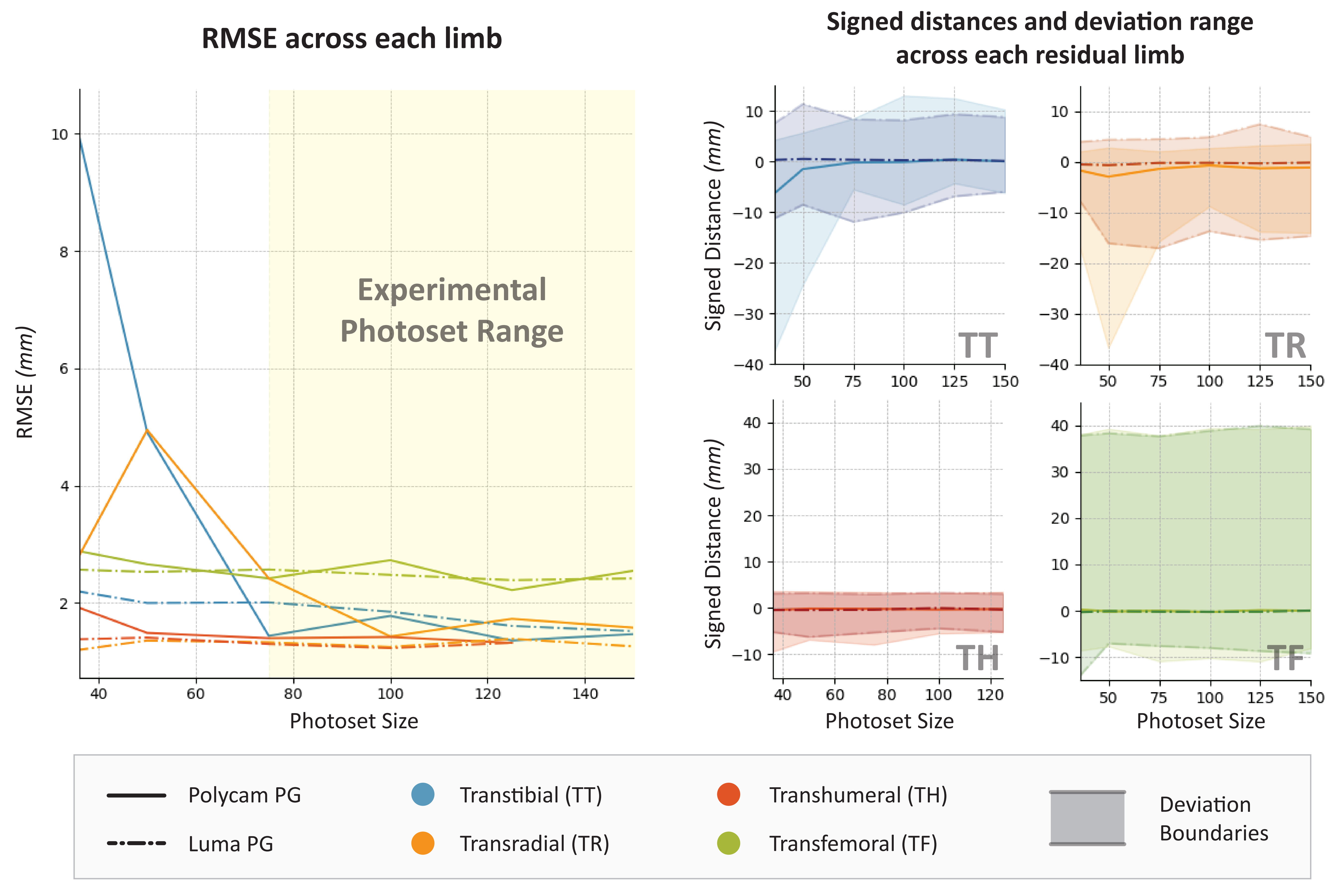
RMSE across each limb
Signed distances and deviation range across each residual limb
Experimental Photoset Range
RMSE (mm)
Photoset Size
Signed Distance (mm)
TT
TR
TH
TF
Polycam PG
Luma PG
Transtibial (TT)
Transradial (TR)
Transhumeral (TH)
Transfemoral (TF)
Deviation Boundaries
