# Supplementary figures and images for: Smartphone scanning is a reliable and accurate alternative to contemporary residual limb measurement techniques
Source: PLoS One. 2024 Dec 2;19(12):e0313542. doi: 10.1371/journal.pone.0313542 (PMC11611122; doi:10.1371/journal.pone.0313542)

# Polycam

# Luma

25  
Photos

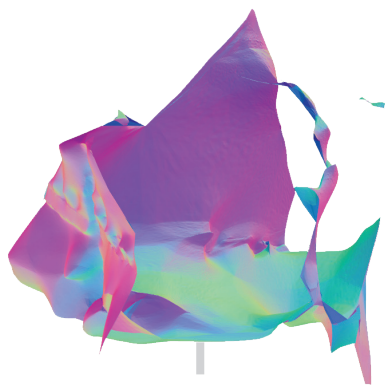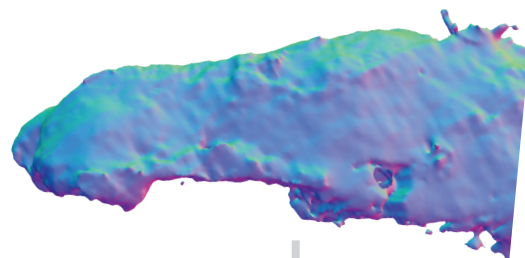

36  
Photos

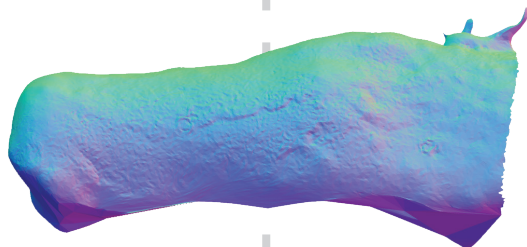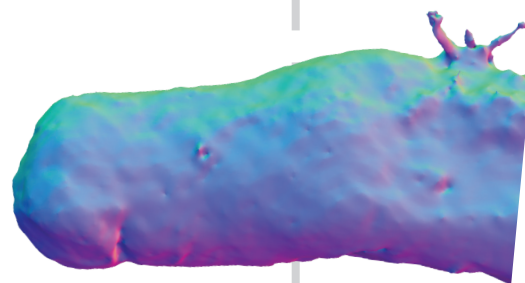

75  
Photos

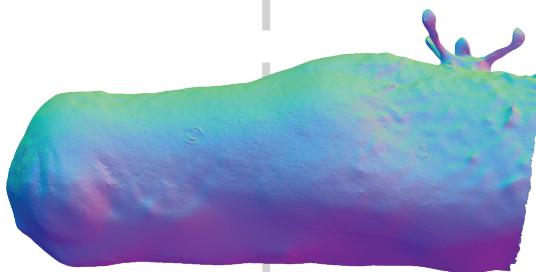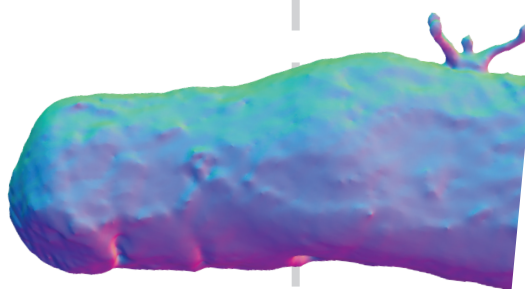

150  
Photos

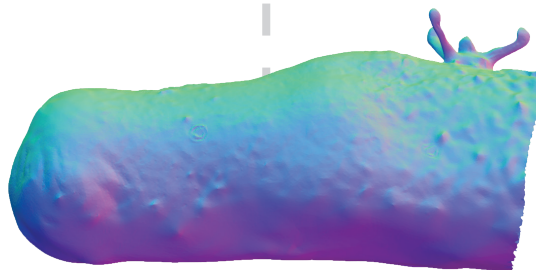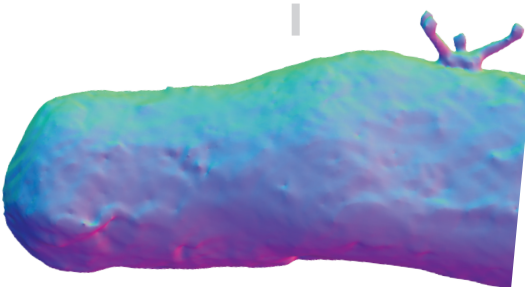

Supplement: S2 Fig — A range of models of a transtibial residuum using the same photoset with varying numbers of photographs made available for the algorithm to use. As demonstrated, there is little difference in surface quality between 75 photographs and 150 photographs, but a significant difference between the other photoset sizes. (PDF) [file pone.0313542.s003.pdf]

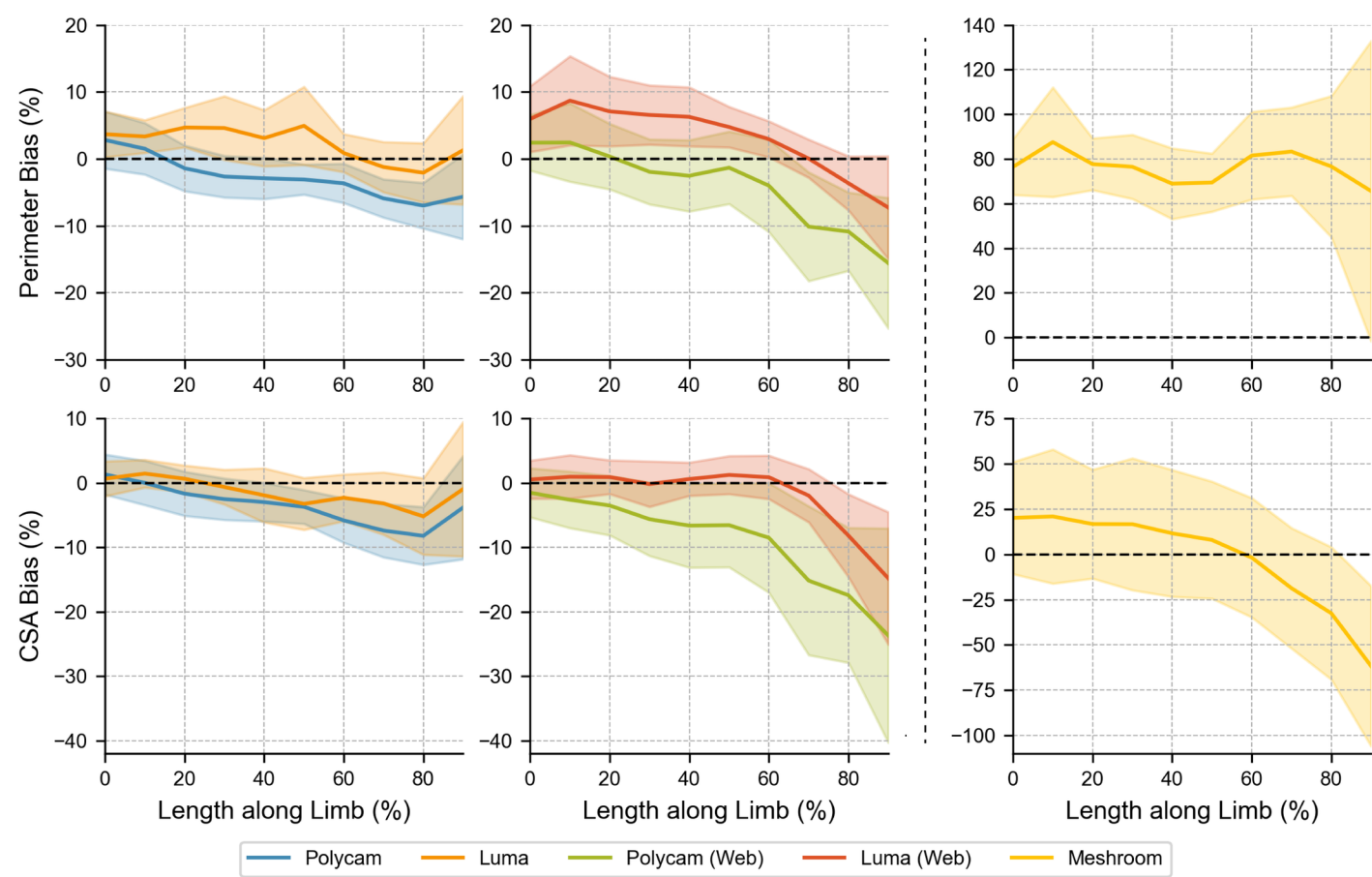

Supplement: S3 Fig — Coloured bands indicate 95% confidence limits. Note that Meshroom necessitates different axis values to the other applications due to it’s significantly worse validity. (PDF) [file pone.0313542.s004.pdf]

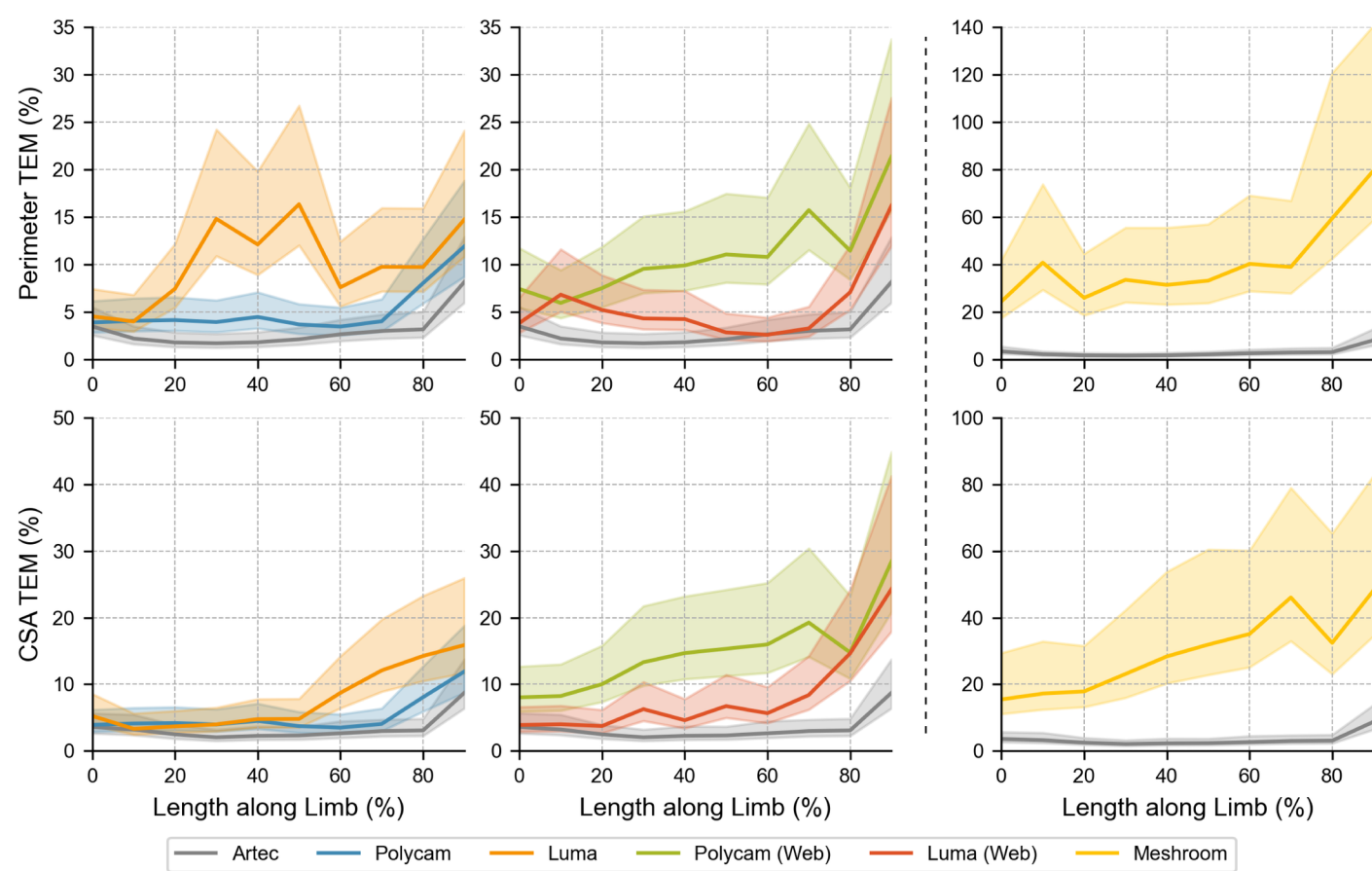

Supplement: S4 Fig — Coloured bands indicate 95% confidence limits. Note that Meshroom necessitates different axis values to the other applications due to it’s significantly worse validity. (PDF) [file pone.0313542.s005.pdf]
