# Supplementary material for: Smartphone scanning is a reliable and accurate alternative to contemporary residual limb measurement techniques
Source: PLoS One. 2024 Dec 2;19(12):e0313542. doi: 10.1371/journal.pone.0313542 (PMC11611122; doi:10.1371/journal.pone.0313542)
Supplement: Attachment — Submitted filename: Response to Reviewers.docx [file pone.0313542.s006.docx]

Response to Reviewers Comments on ‘Remote Scanning of Residual Limbs’

Dear Editor,

We would like to submit a revised version of the manuscript formerly entitled ‘Remote scanning of residual limbs using smartphone technology’ (Ref. No: PONE-D-24-07058), now entitled ‘Smartphone scanning is a reliable and accurate alternative to contemporary residual limb measurement techniques’. Please see our responses below, highlighted in red, to the reviewer’s comments.

We would like to take this opportunity to thank the reviewers for their helpful comments, which we hope you agree have significantly improved the manuscript. We look forward to your decision.

# Response to Reviewer 1

We thank the reviewer for their insightful comments and have implemented proposed amendments into the manuscript where possible, and have performed additional investigations as prompted to validate our findings. With regards to the availability of data used in the study, we are currently in the process of uploading all relevant data captured in the study to the University of Bath Research Data Archive, which will be made publicly available.

**General**: This community typically avoids the terms 'stump' (prefer 'residual limb' or 'residuum') and 'amputee' (prefer person-centred term, 'person with amputation') - recommend changing throughout.
Thank you, we appreciate the importance of inclusive language and have updated the manuscript accordingly. All previous instances of ‘stump’ have been replaced with ‘residual limb’ or ‘residuum’, and all instances of ‘amputee’ have been replaced with ‘person with amputation’ or ‘person with limb difference’, depending upon the context.

**General:** There are also fairly frequent uses of 'better' - could these be replaced throughout with more specific terms? See e.g. point 12 below as one example of a specific alternative, and 21 where it is unclear (Need a better term than better ;))
The two instances of ‘better’ in the original manuscript have been replaced with more informative descriptors. The first was removed entirely during revision of the manuscript. The second instance (lines 431-432 of original manuscript) which previously said ‘… as it produces better surface geometry than Luma’ has been replace with ‘… as it produces fewer defects in low-light areas than Luma’.

**1** The title does not really represent the study, especially the short title; if focusing on 'remote scanning' I would argue the emphasis might be the community-based settings - specific conditions of scanning, like lighting, scanning operated by non-experts/patients/carers, etc. Something around "Accuracy and reliability of smartphone-based 3D scanning apps" might be more appropriate.
We thank the reviewer for highlighting this. We have reframed the article to align better with the evidence we presented and suggest remoted scanning as a potential future direction in the discussion section. This details the suggested methods that would be used to validate such a study, such as using multiple operators in various environments with a consistent limb being scanned to compare the differences between them. The new title is now: ‘*Smartphone scanning is a reliable and accurate alternative to contemporary residual limb measurement techniques*’.

2 Abstract statement "however the question of whether the validity and reliability of the scans produced by these software could meet clinical standards has gone unaddressed." is a bit strong given the introduction references. Perhaps tone this down a little?
The statement in question has been reframed to better reflect the current state of literature in the space: ’Whilst several studies have investigated the potential of privately developed photogrammetry algorithms for capturing residual limbs with clinical accuracy, none to the authors knowledge have explored commercially available software to do the same.’

3 L7: 'oedema' ?
This has been corrected to the appropriate ‘oedema’.

4 L33-7: the Sengeh reference is rather a departure to the concept- in that study MRI was not to capture shape, so reference to earlier review papers (Sanders & Fatone [9], https://doi.org/10.1155%2F2013%2F486146 using MRI to compare PCast and PTB designs) might be more appropriate.
The authors agree with the reviewers assessment of the citation, and as such it has been removed.

5 L49-51: reference 16 also looked at the Sense scanner in models, and https://doi.org/10.1097/JPO.000000000000035 compared Omega, Sense and iSense scanners in living participants.
We thank the reviewer for pointing out this article as an important piece of work on this topic. This study has been integrated into the Introduction section on page 3, referenced as such: ‘Safari et al. explored MRIs potential as a means of measuring residual limb volume by comparing the outcomes of the hands-on and hands-off methods of Patellar Tendon-Bearing (PTB) socket fabrication.’

6 L75-77: references to support the idea of community-based scanning, and considerations specific to this, like environmental factors (lighting, heat) and non-expert operators, as presumably scanning/imaging might require the patient and their carer or family members instead of a trained clinician or community worker? This was pitched in "Technologies to Enhance Quality and Access to Prosthetics & Orthotics: the importance of a multidisciplinary, user-centred approach in https://apps.who.int/iris/rest/bitstreams/1264724/retrieve#page=62)"
We thank the reviewer for another relevant paper suggestion, which has now been included. As raised in suggestion 1, the format of the paper has been restructured to more accurately reflect the evidence provided. The proposed work performed by Dickinson et. Al, has been included to reinforce the presented evidence regarding hobbyist scanners as follows: ‘In further tests by Dickinson et. al, the iSense was compared against the Sense and Omega scanners when scanning both plaster-casts and in-vivo residuums, where it performed the worst of the three across all metrics’.

7: Recruitment details: structure as Inclusion and Exclusion criteria?
The recruitment details have been modified to explicitly name the inclusion and exclusion criteria. This adjustment can be found in lines 110-115 (page 6): ‘Inclusion criteria for the study allowed for any level of major amputation, this being any amputation below wrist or foot disarticulation respectfully. This would allow trends between different amputation levels to be identified, which could inform different guidance on best scanning practices between them. Exclusion criteria necessitated participants be over the age of 18, and for their residuum being scanned to be at least 1 year into maturity.’

8: L126: pitch as a power calculation? Post-hoc would be acceptable!
We thank the reviewer for spotting this oversight and have completed a post-hoc power calculation and included the findings in the recruitment section, lines 118-120 (page 6). We found that the power of this study exceeded 0.99 for all applications tested, including Meshroom. The exact calculation we used can be found below using G*Power (v3.1.9.7), and effect size (ICC) taken from Table 4 of the original manuscript.

9: L159: Were photography conditions standardised in any way? Lighting? Distance? etc.
We have commented in more detail that the subject was photographed from 0.3-0.5 m away, subject to the region of the limb being photographed. It is now explicitly noted that the differences in lighting conditions between the two environments is negligible to the overall study outcomes in lines 143-148 (page 7), stating ‘Due to scheduling conflicts, in vivo scanning of participants was performed between two different environments, so a brief quantitative assessment of the performance of each application in each environment was conducted. A plaster cast model of transtibial residuum was used, pictured in Fig. 2a, and the difference in results between environments was found to be negligible. Consistent lighting was maintained within each environment and between participants’. With regards to the distance, it is later stated in lines 156 - 158 (pages 7-8) that ‘Photographs of the residuum were taken by the same operator approximately 0.5 m from the subject, however this distance decreased to within 0.3 m when capturing photographs below the residuum’.

10: L165-8: Accepting this about number of frames being 'indistinguishable', is there scope to consider testing the number of photos required as an independent variable, vs. accuracy of the result?
We thank the reviewer for this comment, and we conducted additional testing of the existing dataset to validate this statement. We took the largest photosets for each kind of residual limb in the investigation, namely a transtibial, transfemoral, transradial, and transhumeral limb. These were split into separate photosets with quantities of 25, 36, 50, 75, 100, 125, and 150 (where possible). Each of these photosets were then fed back into the Polycam PG and Luma PG algorithms, and the resulting limb scans were compared against a criterion. They were each aligned in Artec Studio and an RMSE analysis was conducted, as was conducted in the main study. This analysis revealed that the size of the photoset had a significant impact upon the accuracy of the scan output, however the difference in RMSE appears to plateau between 75-150 photos in the dataset, supporting our previous assertions. It was interesting to note that the size of the photoset had a significantly greater effect upon Polycam than it did luma. Whilst the size of the photoset did show a small positive correlation with the accuracy of the scan for Luma, the relationship was far smaller than we had previously assumed. This may be due to the incorporation of neural networks in Luma’s design that facilitates more missing angles, as opposed to Polycams more traditional photogrammetry approach. The graph below shows this relationship*:*

This information has been added to lines 163 – 166 (page 8) and to the supplementary material for interested readers, under Supporting Figure 1 (S1 Fig). Alongside these graphs, a visualisation of the generated models is provided as a visual indicator of the quality of the mesh as the photoset used to generate it is increased, also presented in the supplementary material under Supporting Figure 2 (S2 Fig).

11: L186-8: need to manually scale seems to be a major limitation to the method / those devices, and I found the consideration of this as a variable a bit confusing. Could this be simplified, even removed?

We agree that the need for manual scaling is a limitation of the investigated applications. However, we believe the consideration of this variable is important to the paper, and as such have better established the reasoning for including both scaling sets earlier in the paper. This is clarified in lines 133 – 137 (page 7) where these sets are introduced and justified: ‘It is important to note that the use of manual scaling with respect to a reference object may introduce a significant amount of human error, potentially clouding insights into the inherent accuracy of each of the tested scanning applications. Therefore, the study will record two datasets, namely naive and optimal scanning sets.’ The primary focus on the optimal scaling set is reinforced again later in lines 201 – 202 (page 9), where it is said: ‘The naive set and optimal set were analysed separately, and the presented data refers to the optimal dataset unless otherwise stated.’

12: Comment L192-4 on hole filling - could be mentioned in the Discussion as a limitation of these methods, or as part of the argument for investing in Sense or Artec devices?
We appreciate the reviewers concerns relating to access to hole-filling tools and how this may be a limitation in the process outlined in the study. To remedy this, we have mentioned discussed the creation of holed meshed further in the Limitations and Discussions section, outlining the likely causes of such holes, their effect, and how they may be remedied in future studies. Lines 380 – 387 (page 17) read: ‘Another limitation to this study was the need for hole-filling tools to complete meshes. This unavoidably introduces a degree of error, and although the approximated hole-filling geometry likely contributed to the greater RMSE values identified in low-light regions, they are not the sole cause. Instead, these holes are a symptom of poor lighting conditions and insufficient visual data capture from the posterior of the residuums scanned. Improving the lighting conditions under the posterior of the limb, for example by introducing a diffused light source to illuminate it from below, will likely reduce hole instances significantly.’

13: Sorry if I missed it - what was the proximal reference limit / cutoff for volume measurement?
We have added some clarifying statements to where we describe how the residuum is measured to make it clearer, under the Data Processing section, lines 190 – 194 (page 9): ‘A base plane to serve as the proximal reference limit was established perpendicular to the limb’s axis just beneath the reference object, or beneath the knee/elbow joint for transtibial and transradial limbs. The distance between this plane and the end of the limb was measured, and ten sections were created along its length…’.

14: L263-4 are these averages?
Yes, and the text has been altered to make this explicit.

15: L218-7: Report on the mesh size / vertex density generated by each method? And whether this was controlled in any way? Noise may well be related to fine mesh density - coarser mesh looks smoother, up to a point. There's an optimum. Or does this refer to all meshes of same size, so a true comparison?
We thank the reviewer for this suggestion and have implemented it into the paper. Average vertex density for each mesh was determined by dividing the number of vertices in a mesh by the surface area of the mesh. Further analysis was conducted by producing heatmaps in Blender of the vertex density of each mesh; whilst these don’t yield quantitative data, they provide a useful insight into the vertex distribution typical for each application, as shown below:

The vertex density is discussed in further detail and used as supplementary evidence for our findings on the variation in RMSE between the anterior and posterior of the residuum.

16: Not sure what is meant by volatility?
*‘Volatility’* has been changed to a more appropriate ‘volume differences’ on lines 284 – 285 (page 13).

17: L316: 'comfort threshold' might be reading a bit much into that limit - consider toning down?
‘Comfort threshold’ has been altered to a more nuanced discussion based firmly within the findings of the study presented by Lilja et al., which can be found on lines 304 – 305 (page 14): ‘These numbers are in line with the findings of Lilja et al., who documented that a change in residuum volume of 5% is comparable to a single terry sock.’

18: L332 - add 'or muscle tension/activation' to how loose the soft tissue is?
Changed accordingly.

19: L335-9: "higher or lower amputation levels" would be clearer. It's all the body. Assume you mean trans fem or hum vs trans tib or rad?
The passage has been updated to reflect this, and other instances of the previous explanation have been updated accordingly.

20: L378-9: unless you build the scanner and software, or have an open published tool (see e.g. doi:10.1109/ACCESS.2018.2843725, ought to be in Introduction too?) this is always going to be the case?
We agree, and this point has been removed and replaced with a discussion of the problems with scaling, as previously touched on by the reviewer.

21: L388-9: Most smartphone cameras are now very powerful indeed, likely overkill for many applications. How do you envisage 'better' specifically?
It’s been previously noted that smartphone cameras are currently meeting or surpassing the necessary quality for use in clinical environments, hence it may be more suitable to suggest that improvements in the outcomes of smartphone scanners will come from innovations in the software and algorithms used to create such scans, rather than the photographs themselves. This has been discussed and reflected on in detail in the Limitations and Future Directions section.

22: Table 5: Check caption - no numbers in brackets...?
There were several errors here; the number in brackets (that weren’t previously there) were meant to refer to the standard error of the mean differences shown in the table. This has now been rectified and the bracketed numbers have been added.

# Response to Reviewer 2

We thank the reviewer for their helpful comments regarding this paper, which have been very useful in improving the paper. We hope we have addressed the reviewers comments sufficiently.

Reviewer #2: The "Preliminary Investigation" section presents the research rationale in a somewhat disorganized manner. I recommend including a flowchart to visually guide the reader through the research logic and results, which would clarify the investigative process.
We thank the reviewer for their insight regarding the presentation of the preliminary investigation section, and how it may be confusing to the reader. Whilst a flowchart may be useful, the number of figures included in the article is already significant, therefore, instead improvements to the explanation of the preliminary investigation itself have been made to make it more straightforward. The description of the preliminary investigation now reads as such on lines 88 – 99 (page 5): Simple qualitative assessments were used for the initial filtering of suitable scanning applications, by using visual assessment of mesh and texture data produced from a variety of household objects. These objects were chosen for their unique geometrical features that would test the limits of the scanning applications in question, and establish their abilities beyond the comparatively simple geometry of a residual limb. Two such examples of scanning objects used were a coffee mug for testing through-hole and blind-hole generation, and a leafy plant for thin element generation. Each of these geometrical features offer opportunities for different kinds of mesh distortion to reveal itself that may otherwise be difficult to identify when scanning simple residual limbs. The quality of meshes compared to one-another was visually evident, reducing application choices to Polycam, Luma, and Meshroom.’*.*

Lines 103-104 mention that various household objects, including coffee cups and plants, were used to generate models through the applications, followed by an evaluation of scan quality. However, the selection criteria for these objects are unclear. What was the rationale behind choosing these particular items? Please provide justification for their selection.

The rationale behind the selection of these specific objects, with regards to their unique geometry as to test the limits of each scanning applications abilities, has been outlined in the article. The coffee cup for example was used for its blind-hole and through-hole features, and the plant was used to determine how the scanners modelled thin elements, as mentioned in the response above.

In Lines 124-125, the paper references lower and upper limb amputees. Could you clarify whether these conditions influence the scanning outcomes? It would be beneficial to explore how such factors might affect the accuracy or quality of the scans.
We thank the reviewer for this comment and agree that the original manuscript could have made clearer the influence of amputation level on scan outcomes. These have been made more explicit in the discussion section, in which we discuss the differences in results between the various amputation levels and how said results may inform future scanning practices. Predominantly, the conclusion arrived at from the results is that the scanning process outlined is likely to produce more reliable and accurate results for upper limbs than lower limbs. This is explained in lines 320-328 (page 15): ‘Soft tissue can hang differently between scans depending on multiple factors, including the participant posture, the angle of their limb, and the level of muscle activation in the residuum at time of scanning. This problem may be exacerbated in clinical practice where the time between scans could be a matter of weeks rather than minutes. This is partly the reason why higher amputation levels exhibit greater RMSE values compared to limbs of lower amputation levels with comparable volume. Transfemoral and transhumeral residuums store a greater proportion of soft tissue, hence the potential for a shift in the soft structure increases’*.* This is further elaborated on in lines 332 – 338 (page 15): ‘However, this means the posterior remains in shadow when lifted horizontally, despite efforts to ensure uniform lighting. This difficulty is again exacerbated with lower-limb amputations where the limb remains closer to the floor, making uniform coverage potentially difficult. These factors suggest that smartphone scanning may be best suited to patients with upper-limb amputations, and that improvements to the scan collection process may be necessary when dealing with lower-limb amputations.’

Mobile scanning technology is already widely available, and the accuracy of these products has been well-documented. Please elaborate on the innovations of this study, particularly in addressing clinical problems and advancing scanning technology beyond existing solutions.
Whilst previous studies may have documented the potential of using photographs captured with a smartphone in a traditional photogrammetry pipeline for deriving residuum measurements, none to the authors knowledge have discussed the use of commercially available and accessible applications to do the same. This presents a significant insight into how such methods could be implemented into clinical practice swiftly with minimal investment.
